# *KRAS* Mutations in Primary Colorectal Cancer Tumors and Related Metastases: A Potential Role in Prediction of Lung Metastasis

**DOI:** 10.1371/journal.pone.0008199

**Published:** 2009-12-18

**Authors:** Paloma Cejas, Miriam López-Gómez, Cristina Aguayo, Rosario Madero, Javier de Castro Carpeño, Cristóbal Belda-Iniesta, Jorge Barriuso, Víctor Moreno García, Javier Larrauri, Rocío López, Enrique Casado, Manuel Gonzalez-Barón, Jaime Feliu

**Affiliations:** 1 Department of Medical Oncology, La Paz University Hospital, Madrid, Spain; 2 Biostatistics Unit, La Paz University Hospital, Madrid, Spain; 3 Department of Pathology, La Paz University Hospital, Madrid, Spain; 4 Department of Medical Oncology, Infanta Sofía Hospital, Madrid, Spain; University of Barcelona, Spain

## Abstract

**Background:**

*KRAS* mutations in colorectal cancer primary tumors predict resistance to anti-Epidermal Growth Factor Receptor (EGFR) monoclonal antibody therapy in patients with metastatic colorectal cancer, and thus represent a true indicator of EGFR pathway activation status.

**Methodology/Principal Findings:**

*KRAS* mutations were retrospectively studied using polymerase chain reactions and subsequent sequencing of codons 12 and 13 (exon 2) in 110 patients with metastatic colorectal tumors. These studies were performed using tissue samples from both the primary tumor and their related metastases (93 liver, 84%; 17 lung, 16%). All patients received adjuvant 5-Fluorouracil-based polychemotherapy after resection of metastases. None received anti-EGFR therapy. Mutations in *KRAS* were observed in 37 (34%) of primary tumors and in 40 (36%) of related metastases, yielding a 94% level of concordance (kappa index 0.86). Patients with primary tumors possessing *KRAS* mutations had a shorter disease-free survival period after metastasis resection (12.0 vs 18.0 months; P = 0.035) than those who did not. A higher percentage of *KRAS* mutations was detected in primary tumors of patiens with lung metastases than in patients with liver metastases (59% vs 32%; p = 0.054). To further evaluate this finding we analyzed 120 additional patients with unresectable metastatic colorectal cancer who previously had their primary tumors evaluated for *KRAS* mutational status for clinical purposes. Separately, the analysis of these 120 patients showed a tendency towards a higher degree of *KRAS* mutations in primary tumors of patients with lung metastases, although it did not reach statistical significance. Taken together the group of 230 patients showed that *KRAS* was mutated significantly more often in the primary tumors of patients with lung metastases (57% vs 35%; P = 0.006).

**Conclusions/Significance:**

Our results suggest a role for *KRAS* mutations in the propensity of primary colorectal tumors to metastasize to the lung.

## Introduction

Colorectal cancer (CRC) is one of the most common malignancies and one of the leading causes of cancer-related death in developed countries [1]. Distant metastasis is the main cause of death in CRC patients. Depending on the stage of the primary tumor, liver metastases occur in 20% to 70% of patients, and lung metastases in 10% to 20% of patients [2]. Surgical resection remains the only potentially curative option for patients with metastatic CRC. However, curative resection is possible in less than 25% of patients with stage IV disease [3], and less than 5% of patients with unresectable metastatic CRC are alive after 5 years. Major efforts are being made to improve the prognosis for patients with metastatic CRC, especially in the development of new therapeutic strategies. The Epidermal Growth Factor Receptor (EGFR) signalling pathway has become a key target for therapeutic intervention because two monoclonal antibodies directed against EGFR have become important tools in the management of advanced disease: cetuximab and panitumumab [4], [5]. EGFR activates proliferative and antiapoptotic signalling pathways, such as the phosphatidylinositol 3′ kinase/Akt and Ras/Raf/mitogen-activated protein kinase (MAPK) pathways [6]. Aberrant activation of the EGFR pathway in CRC could be caused by either EGFR overexpression or mutational activation of downstream elements of the EGFR pathway [7].

*KRAS* is a small GTP-binding protein that transduces signals from activated cell surface receptors to the nucleus. Constitutive *KRAS* activation by point mutations in codons 12 and 13 of exon 2 has been described as an important cause of EGFR pathway overactivation [7], [8]. The incidence of *KRAS* mutations in colorectal tumors ranges from 35% to 45% [9], and *KRAS* mutations seem to occur early in carcinogenesis [10]. Accordingly, a high degree of concordance in *KRAS* mutational status between primary tumors and their related liver metastases has been reported [11], [12]. Recent data have demonstrated an association between *KRAS* mutational status in the primary tumor and resistance to cetuximab and panitumumab in patients with metastatic CRC [13], [14]. However, the association between *KRAS* mutational status and prognosis is controversial for patients with metastatic CRC that have not been treated with anti-EGFR antibodies, with some studies reporting a link between *KRAS* mutations and poor prognosis [15] and some reporting no association [12]. Interestingly, the largest multicentre study conducted on the association between *KRAS* mutation and prognosis, which included 3439 CRC patients, showed that the presence of a glycine-to-valine mutation at codon 12 of *KRAS* significantly decreased progression-free and overall survival rates irrespective of the treatment received [16].

We sought to elucidate the correlation between *KRAS* mutational status, clinicopathologic factors, prognosis, metastasis pattern and concordance between the primary tumor and matched metastases in patients with metastatic CRC.

## Results

### Patient Characteristics

We retrospectively analysed specimens from 110 primary tumors and 110 corresponding metastatic sites for the presence of *KRAS* mutations in codons 12 and 13. The most common metastatic site was the liver, which was the metastatic site in 93 samples (84%). The lung was the metastatic site in the remaining 17 samples (16%). Metastases appeared synchronously with the primary lesion in 57 cases and metachronously in 53 cases. The primary tumor site was the colon in 79 patients and the rectum in 31 patients. The patient group included 32 women and 78 men. Median age was 64 years (29-86). All cancers were adenocarcinomas and were graded according to WHO criteria (Table 1).

**Table 1:** Patient characteristics.

Characteristic	Value
Total number of primary tumors	110
Total number of metastatic samples	110
Liver	93
Lung	17
Age, median (range)	64 (29–86 yrs)
Gender (male/female)	78/32
Histological Grade	
1	2
2	98
3	10
Primary tumor site (colon/rectum)	79/31
Primary stage (WHO classification)	
I	3
II	20
III	28
IV	59
Adjuvant treatment schedule	
5-Fluorouracil/Leucovorin	36
Oxaliplatin/5-Fluorouracil/Leucovorin	46
Irinotecan/5-Fluorouracil/Leucovorin	28
*Neoadjuvant treatment schedule*	
Radiotherapy (45 Gy)/5-Fluorouracil/Leucovorin	8
Radiotherapy (45 Gy)/Raltitrexed/Oxaliplatin	15
Radiotherapy (45 Gy)/Capecitabine/Oxaliplatin	8
Synchronous/Metachronous metastases	57/53

### *KRAS* Mutations and Histopathological Parameters

Mutations in *KRAS* were detected in 37 (34%) primary tumors and in 40 (36%) related metastatic lesions, with a 94% grade of concordance between primary and metastatic samples (kappa index 0.86). Discordance was observed in 7 patients. Five patients had *KRAS* mutations in the metastases (four with liver metastases and one with lung metastases) and wild type *KRAS* in the primary tumor, whereas the two other patients had *KRAS* mutations only in the primary tumor (1 with liver metastases and 1 with lung metastases).

There was little variance between the mutation type present in the primary tumor and its metastases: all but four patients had the same mutation in both the primary and the metastatic samples. The types of mutations are summarized in Table 2. There was no relationship between the type of mutations and any clinicopathological parameter.

**Table 2:** Codon distribution of specific *KRAS* mutations.

Mutations	Primary tumors	Liver metastases	Lung metastases
Gly12Ala	2	2	1
Gly12Asp	16	12	4
Gly12Cys	2	2	0
Gly12Ser	0	0	1
Gly12Val	11	9	2
Gly13Asp	6	5	2
Total	37	30	10

No associations between *KRAS* mutations and histopathological characteristics were observed (Table 3).

**Table 3:** Relationship between *KRAS* mutational status and tumoral variables measured in primary tumors.

	Mutated KRAS		WT *KRAS*		P value
	N°	%	N°	%	
Gender					0.376
Male	24	31%	54	69%	
Female	13	41%	19	59%	
Age					1.000
>60	24	33%	48	67%	
<60	13	34%	25	66%	
Tumor location					0.825
Rectum	11	36%	20	64%	
Colon	26	33%	53	67%	
Obstruction					0.616
Yes	6	27%	16	73%	
No	31	35%	57	65%	
Perforation					0.176
Yes	4	67%	2	33%	
No	33	32%	71	68%	
Primary tumor stage					0.970
I	1	33%	2	67%	
II	6	30%	14	70%	
III	9	32%	19	68%	
IV	21	36%	38	64%	
Metastasis presentation					0.546
Synchronous	21	37%	36	63%	
Metachronous	16	30%	37	70%	
Lymphatic invasion					0.095
Yes	9	53%	8	47%	
No	28	30%	64	70%	
Venous invasion					0.616
Yes	6	27%	16	73%	
No	31	36%	56	64%	
Perineural invasion					0.760
Yes	5	39%	8	61%	
No	32	33%	64	67%	

### *KRAS* Mutations and Prognosis

**Figure 1:**
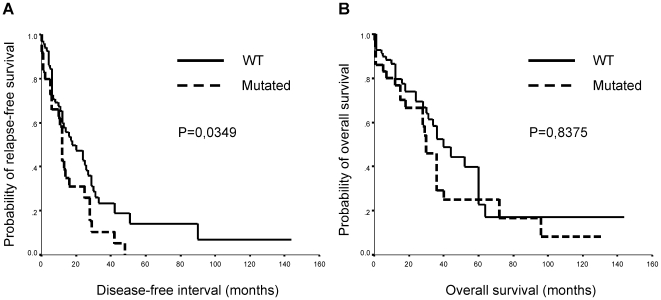
Kaplan-Meier survival analysis. Curves for disease-free survival (A) and overall survival (B) according to *KRAS* status.

Our results show lower rates of disease-free survival after metastasis resection in patients whose tumors had a *KRAS* point mutation in the primary tumor; median disease-free survival was 12 months in patients with *KRAS* mutations in their tumors and 18 months in those without *KRAS* mutations (p = 0.035) (Figure 1). The multivariate analysis showed that the most significant independent predictor for disease-free survival was *KRAS* mutational status (multivariate HR = 2.068; 95% CI, 1.136–3.766, P = 0.018) (Table 4). No association was found between overall survival and *KRAS* mutational status (figure 1).

**Table 4:** Multivariate disease-free survival analysis.

	HR	95% CI	P
Primary tumor *KRAS* status	2,068	1,136–3,766	0,018
Primary tumor stage	1,165	0,868–1,563	0,310
Metastases size	0,999	0,899–1,109	0,981
Total number of metastases	1,018	0,876–1,184	0,813
Neoadjuvant treatment	1,457	0,770–2,755	0,247
Metastasis site	0,582	0,274–1,237	0,159

Abbreviations: HR: Hazard Ratio. CI: Confidence Interval.

### *KRAS* Mutations and Metastatic Site

Mutated *KRAS* was detected in a higher proportion of primary tumors of patiens with lung metastases than in those with liver metastases (59% vs 32%, P = 0.054). When stratified by origin of the primary tumor (colon or rectum), of the 10 *KRAS*-mutated primary tumors of patients with lung metastases, 7 were colonic in origin (70%) whereas the other 3 originated in the rectum (30%). Statistical analysis revealed that colonic primary tumors of patients with lung metastases were significantly more likely to have *KRAS* mutations than colonic primary tumors of patients with hepatic metastases (P = 0.046), whereas rectal primaries had a similar frequency of *KRAS* mutations in patients with lung or liver metastasis (Table 5). To further investigate the presence of *KRAS* mutations as a predictor of lung metastasis, we also examined a series of 120 patients with unresectable metastatic CRC. The primary tumors of these patients had been analysed for the presence of *KRAS* mutations by a central laboratory prior to cetuximab administration. Of the 120 metastatic patients, 86 had liver metastases and 34 had lung metastases. In addition, there was a tendency towards a higher proportion of *KRAS* mutations in primary tumors of patients with lung metastases than in those with liver metastases, although it did not reach statistical significance (56% vs 38%; P = 0.1027). When we analysed the whole series of 230 patients, the frequency of *KRAS* mutations in the primary tumors of patients with lung metastases was significantly higher as compared with liver metastases (57% vs 35%; P = 0.006). When patients were stratified by primary tumor origin, only patients with colonic primary tumor origin showed a significantly higher frequency of *KRAS* mutations in primary tumors of patients with lung metastases as compared with those with liver metastases (59% vs 34%; P = 0.019) (Table 6).

**Table 5:** *KRAS* mutational status in the primary tumor according to the metastatic site (series of 110 patients).

	Metastatic site	WT	Mutated	Total	P
*KRAS* (colon)					0,046
	liver	47 (69%)	21 (31%)	68	
	lung	4 (36%)	7 (64%)	11	
*KRAS* (rectum)					0,653
	liver	16 (64%)	9 (36%)	25	
	lung	3 (50%)	3 (50%)	6	

**Table 6:** *KRAS* mutational status in the primary tumor according to metastatic site (whole series of 230 patients).

	Metastatic site	WT	Mutated	Total	P
*KRAS* (colon)					0,019
	liver	91 (66%)	46 (34%)	137	
	lung	12 (41%)	17 (59%)	29	
*KRAS* (rectum)					0,304
	liver	25 (60%)	17 (40%)	42	
	lung	10 (45%)	12 (55%)	22	

## Discussion

The prognosis for patients with metastatic CRC has improved since the introduction of novel therapeutic agents such as anti-EGFR antibodies [17]. This therapeutic success highlights the importance of counteracting the EGFR pathway to control advanced disease. Aberrant activation of the EGFR pathway sometimes occurs by mutational activation of *KRAS*. Recent clinical trials have shown that the presence of activating mutations in *KRAS* identifies patients who are non-responders to cetuximab [13] or panitumumab [14]. In fact, based on these results, mutational analysis of *KRAS* is now recommended by The US Food and Drug Administration (FDA) prior to cetuximab or panitumumab administration to patients with metastatic CRC. Mutational analysis of *KRAS* is commonly performed on the primary tumor because it is often the only available tissue. Moreover, the value of performing this analysis on the primary tumor is further supported by evidence that *KRAS* point mutations occur early in CRC carcinogenesis [10]. However, recent data have demonstrated an increased frequency of mutations in lymph node metastases as compared with their related primary tumors [18]. With this in mind, the potential need for rebiopsy and analysis of *KRAS* in the metastases has been suggested [9]. Other recent studies have reported a high degree of concordance in *KRAS* mutational status between primary tumors and their related liver metastases [11], [12], [19]. The results from our series of 110 patients are in complete agreement with these reports, both in the frequency of *KRAS* mutations in primary tumors, which was 34% in our study, and in the high degree of concordance between primary tumors and their related metastases. All of these findings confirm that analysis of *KRAS* mutational status in the primary tumor is an adequate surrogate marker of *KRAS* mutational status in metastases. Our study includes an analysis of both liver and lung metastases. As far as we know, this is the first study to analyse primary colorectal tumors and their related lung metastases and correlated *KRAS* mutational status based on both clinicopathological features and survival data. The liver and the lung are both common sites of CRC metastases. Secondary to their respective anatomical blood vessel distribution, lung metastases are more common with rectal cancers and liver metastases are more frequent with colon cancers. However, some colon cancer patients experience lung metastases without evidence of previous liver metastases. This unexplained anatomical pattern of metastasis, observed with increasing frequency due to more accurate diagnoses based on highly efficient CT scans [20], suggests a peculiar biological mechanism of carcinogenesis or a special susceptibility of the lung parenchyma to tumors in these patients. Our results demonstrate a higher percentage of *KRAS* mutations in primary tumors of patients with lung metastases as compared with those with liver metastases (59% vs 32%). Moreover, when we stratified our results based on the primary tumor site, only tumors of colonic origin had a significantly higher frequency of *KRAS* mutations in primary tumors of patients with lung metastases. By contrast, rectal primary tumors showed a similar frequency of *KRAS* mutations in patients with lung metastases than in those with liver metastases. To further analyze this finding we studied an independent series of primary tumors from patients with unresectable metastatic CRC and examined the relationship between *KRAS* mutational status in the primary tumor and the site of tumor metastasis. In these patients, *KRAS* mutational status in the primary tumor was previously analysed for clinical purposes prior to cetuximab administration. Statistical analysis of the whole series (230 patients) revealed that *KRAS* is more frequently mutated in the primary tumors of patients with lung metastases compared with patients with liver metastases (57% vs 35%, P = 0.006). When the results are stratified according to the site of the primary tumor, the analysis we performed including all 230 patients confirmed the results we obtained with the smaller sample of 110 patients: that the significantly increased frequency of *KRAS* mutations in primary tumors in patients with lung metastases was restricted to those primary tumors that originated in the colon. This finding suggests that EGFR pathway activation may allow colonic tumor cells to nest preferentially in the lung parenchyma avoiding an initial step of liver metastasis. A previous study that evaluated *KRAS* status in primary colorectal tumors and non-matched liver and lung metastases showed concordant results with our study and revealed a higher incidence of *KRAS* mutations in lung metastases than in liver metastases (57% vs 50%) [21]. However, in that study, a major difference in *KRAS* activation between the primary and metastatic tumors could have been masked by the absence of related primary and metastatic samples. This finding has clinical importance because it may allow the identification of patients who are more likely to develop lung metastases based on *KRAS* analysis of the primary tumor. These patients should potentially receive a more thorough clinical workup, including a thorax scan, which is not always included in the standard clinical workup for CRC.

Our results also show that the presence of *KRAS* mutations is an independent prognostic factor in the prediction of disease-free survival (reaching statistical significance). Constitutive activation of the Ras-Raf-MAP-kinase pathway is known that confers proliferative and disseminative advantage to tumor cells. However, controversy exists regarding the prognostic role of KRAS mutations in CRC. The biggest clinical trial designed to analyse the prognostic value of *KRAS* status was the RASCAL study, which showed that a glycine-to-valine mutation in codon 12 increased the risk of recurrence and death by 30%, irrespective of the type of therapy administered [16]. However, a recent study performed with a small number of patients did not reproduce this result and did not demonstrate any influence of *KRAS* mutations on survival [12]. Thus, the prognostic value of *KRAS* mutational status remains controversial. Our results are in agreement with the RASCAL study and suggest a poorer prognosis for patients with *KRAS*-mutated primary tumors, with a significant association between *KRAS* mutational status and a shorter disease-free survival. By contrast, no association was found between *KRAS* status and overall survival. This can be explained by the administration of chemotherapy after relapse. The multivariate analysis shows that the presence of *KRAS* mutations is an independent prognostic factor in the prediction of disease-free survival (reaching statistical significance), thus highlighting the relevance of *KRAS* mutational status in the present series of patients. Regarding a potential influence of the mutation type on prognosis, our results, in contrast to the RASCAL study, show no differences with respect to mutation type. Of course, the potential value of *KRAS* mutations as a prognostic factor shown in this study must be taken cautiously, due to the limited and particular series of patients evaluated.

In summary, our findings suggest that *KRAS*-mutated primary CRC tumors tend to metastasize more frequently to the lung and are associated with a higher rate of relapse after resection of the metastases. This finding could be of clinical interest with regard to the follow-up of patients with *KRAS*-mutated primary CRC tumors, and thus merits further investigation.

## Methods

### Ethics Statement

The Ethical Committee of the La Paz University Hospital in Madrid, Spain, approved the current study. All patients were informed and consented in writing.

### Eligible Patients

One hundred and ten patients with metastatic CRC with available primary tumor and paired metastatic specimens were selected from a database of patients from La Paz University Hospital in Madrid. This study was approved by the hospital's ethics committee. The patients were diagnosed with metastatic CRC between 1997 and 2007. Of the 326 cases of metastatic CRC diagnosed during this time period, study inclusion was limited to those with curative metastasis resection, available tissue specimens (both from the primary tumor and related metastases), available follow-up data after metastasis removal, and successful *KRAS* mutational status analysis. The median follow-up after surgery was 10 months (range: 0–144 months). All tumors were histologically confirmed to be colorectal adenocarcinomas. In addition, pathology reports included information on the tumor site, tumor size, pathological stage, tumor grade (based on the degree of histological differentiation), presence or absence of lymph node metastasis, tumor growth pattern (infiltrative or expansive), presence or absence of vascular and lymphatic invasion, and presence or absence of tumor lymphocytic infiltrates. The primary stage has been classified into 4 stages according to WHO criteria. The number and size of metastases were determined by diagnostic imaging, either with abdominal and pelvic computed tomography or with magnetic resonance imaging. Metastasis resection was performed when there was no evidence of extra-hepatic disease, ensuring enough functioning residual liver after resection (approximately 25%–30% of normal liver volume) to provide hepatic function postoperatively, adjusted based on existing liver disease such as cirrhosis or hepatitis. Metastatic samples were obtained from either the liver or the lung. These metastatic samples were obtained from either synchronous or metachronous lesions. Patients received one of three different chemotherapy regimens after metastasis resection (described in Table 1).

After the discovery of the discordance between *KRAS* mutations in liver and lung metastases, an additional series of 128 metastatic CRC patients (86 with liver metastasis, 34 with lung metastasis, and 8 with other types of mutations (these last 8 patients were not included in the statistical analysis) from La Paz Hospital were prospectively collected between 2007 and 2009, to validate the hypothesis of a higher rate of *KRAS* mutations in patients with lung metastases. These patients had unresectable metastatic disease. *KRAS* status in the primary tumor was determined for clinical purposes prior to cetuximab administration. The only clinicopathological data collected for these patients was the site of the primary tumor (colon vs rectum). *KRAS* analysis was performed using the TheraScreen K-RAS mutation test kit (DXS Diagnostic Innovations) by an independent central laboratory in Madrid, following the manufacturer's instructions.

### DNA Extraction

Paraffin-embedded primary tumor specimens (n = 110) and metastatic tumor specimens (n = 110) containing at least 70% tumor cells were selected for each patient. Tissue blocks were macro-dissected using a safety blade when samples were less than 70% enriched with tumoral cells. Metastatic tumors were located in the liver (n = 93) and the lung (n = 17). DNA was isolated from 15 7-µm paraffin sections. Tissue sections were deparaffinated with xilol and rehydrated with downgraded alcohols. Tissues were digested with Proteinase K, and DNA was isolated using a MasterPure DNA Kit (Epicentre, Biotechnologies). In each instance, negative controls were amplified by PCR and included in the experiment.

### *KRAS* Mutation Analysis

A polymerase-chain reaction (PCR) was performed to amplify 139 bp of exon 2 in KRAS using specific primers (kindly donated by Dr. Laurent-Puig's laboratory) and under PCR conditions described previously [15]. PCR primer sequences/conditions are available upon request. The efficiency and quality of the amplification PCR were confirmed by running the PCR products on a 2% agarose gel. A negative control containing all the components of the PCR except the template was included in each PCR reaction. DNA Amplified products were purified using a QuickStep TM 2 96–Well PCR Purification Kit (Edge BioSystems, Gaithersburg, MD), according to the manufacturer's instructions. Amplification products were bidirectionally sequenced via the fluorescence dye terminator method in a multi-capillary DNA sequencer using the services of the scientific park of Madrid (Madrid, Spain). Presence of mutation was accepted when it chromatographic peak height was 25% or higher the peak of the wild type reference.

### Statistical Analyses

Fisher's exact test was used to examine the association between *KRAS* mutational status and various clinicopathological features. Concordance between the primary tumor and related metastases was analysed using the kappa index, and discordance was analysed using the McNemar test of symmetry. Disease-free survival and overall survival analyses were calculated according to the Kaplan Meier method, and survival curves were compared using the log-rank test. Disease-free survival was defined as the time from surgical resection of the metastases until the time of documented tumor progression or death. A Cox proportional hazards multivariate regression analysis was performed. P<0.05 was considered significant. Statistical analyses were performed using SPSS version 12.0 (Chicago, IL, USA).
